# Do media coverage of suicides and search frequency on suicides predict the number of tweets seeking others for a suicide pact?

**DOI:** 10.3389/fpsyt.2023.1260567

**Published:** 2023-09-29

**Authors:** Sang Yup Lee

**Affiliations:** Department of Communication, Yonsei University, Seoul, Republic of Korea

**Keywords:** suicide pact, Twitter, social media, search frequency, celebrity suicide, media coverage of suicides

## Abstract

We examined whether media coverage of suicides and frequencies of searching for suicide methods or suicide pacts predicted the number of users posting tweets seeking others for a suicide pact. Analyses of 6,119 tweets containing “suicide pact” posted on Twitter during a 6-month period revealed that the number of users posting tweets seeking others for a suicide pact had a positive association with media coverage of celebrity suicides, but not with that of suicide pact victims, and a greater positive association with the search frequency for suicide methods than for suicide pacts. We found that the search frequency on suicide methods was positively associated with media coverage of celebrity suicides, while that on suicide pacts was more strongly related to media coverage of suicide pacts.

## 1. Introduction

In the past, suicide pacts, which are defined as an agreement between two or more people to die together by suicide (1), accounted for only a small fraction of all suicides (2). However, the number of suicides due to suicide pacts appears to have increased over time, especially in some Asian countries such as Japan and South Korea (1, 3, 4). For example, a news article estimated that suicide pacts account for about one-third of the total suicides in South Korea (5).

In the past, suicide pact was formed in offline settings between acquaintances such as family members or couples, yet recently with the development of communication technologies, suicide pacts are more commonly formed in online settings in most case between strangers (6). The devastating point is that a suicide pact formed on the Internet is frequently leads to real suicides (2, 7). Accordingly, it is critical to monitor suicide pacts formed on the Internet and prevent individuals from committing suicide through such a suicide pact.

With the popularity of social media such as Facebook and Twitter, many people tend to use them to seek others to make a suicide pact (4, 8–10). Twitter is known to be a particularly popular platform in this respect, because of such features as greater anonymity and user-created content being more publicly consumed by than that on other platforms (8). Twitter is more frequently used for making suicide pacts in certain countries, such as South Korea and Japan, because as a foreign platform, it is less influenced by domestic laws and regulations concerning the posting of pro-suicide information (8). Several cases of suicide pacts formed via Twitter have been reported in these countries (11, 12). Lee and Kwon (8) found that many South Koreans were using Twitter for this purpose: on average, 21 users posted such tweets per day.

Posting a tweet to seek others for a suicide pact exerts a fatal influence on not only the person who created it but also other users who search for these tweets, that is, those who are unwilling to post such tweets for themselves, but want to join others for a suicide pact. When users can easily search for suicide pacts and discover others in similar circumstances, they are likely to be more inclined to die by suicide or join a pact (8, 13).

To mitigate the negative effects of tweets seeking others for a suicide pact posted on social media such as Twitter, it is important to understand what influences or stimulates such posting behavior, which will also enable a better understanding of when people want to form a suicide pact, especially on the Web.

In this study, we examine how the number of users posting such tweets is associated with the media coverage of suicides and the frequency of searching for suicide-related terms on Internet search engines. For media coverage of suicides, in this study, we focus on the media coverage of celebrity suicides and suicides as a result of a suicide pact. First, a number of previous studies found that media coverage of celebrity suicide tends to lead to copycat suicides (14–19), mainly due to individuals’ identification with a celebrity. According to identification theory (20), an individual tends to identify with a celebrity because that person perceives them as somehow superior; this vertical identification then leads some to copy that celebrity’s behavior, including suicide. This suggests that following media coverage of celebrity suicide, more people may post tweets seeking others for a suicide pact in an attempt to end their lives.

On the other hand, the horizontal identification principle posits that individuals are also likely to identify with those who are in similar circumstances (16). This suggests that posting a tweet seeking others for a suicide pact can be stimulated by media coverage of suicide pact victims: through such reports, people realize there are others in a similar situation who considered forming a suicide pact and successfully ended their lives.

In this study, we also examine how the frequency of searching suicide related terms, that is, “how to die by suicide” and “suicide pact” on online search engines can predict such posting behavior on Twitter. Those considering suicide are likely to search for “how to die by suicide,” to find likely methods (21). As a result, they are likely to find information about suicide pacts and that others can be sought for a pact through Twitter. On the other hand, those considering a pact as a means of suicide are likely to search for “suicide pact” to find out how. Some search results may direct them to Twitter for this purpose, which could lead to more users posting tweets seeking a suicide pact.

Finally, we examine how the search frequency on “how to die by suicide” or “suicide pact” is associated with media coverage of celebrity suicides or suicide pact victims. As the vertical and horizontal identification principles suggest, more individuals could consider attempting suicide following such media coverage, suggesting more would seek information about “how to die by suicide” or “suicide pact.”

## 2. Methods

### 2.1. Data

#### 2.1.1. Tweets seeking others for a suicide pact

We used all Korean tweets containing the term “suicide pact” collected using Twitter application programming interfaces (APIs), which are computational tools that help developers to acquire tweets data, from October 16, 2017 to April 28, 2018. Data were missing for 15 of the 195 days in the data collection period (i.e., October 25, November 13, 15, 30, December 13–14, February 14–16, March 24–26, 30–31, and April 1) due to network connection issues. Excluding these days from the final analysis, the data set thus contained tweets about suicide pacts on 180 days.

We used Python, a computer programming language, to identify tweets seeking others for a suicide pact from all those collected. For this, we first identified the part-of-speech of each word used in a tweet using the Twitter part-of-speech (POS) tagger offered in the KoNLPy module in Python (22). To alleviate the out of vocabulary problem when using the POS tagger, we also extracted Korean nouns using the “kornounextractor” module provided in Python (23). With frequency analysis, we calculated the frequencies of noun and verb words used in the tweets. The frequency analysis revealed noun words reflecting methods of suicide, places of the intended suicide pact, contact methods, and verb words that could be used to seek others for a suicide pact, e.g., “seek,” “join,” “send (a message),” and “leave.” Based on these words, to identify all other noun words related to methods of suicide, places of the intended suicide pact, contact methods, and verb words related to seeking others for a suicide pact, we used the Word2vec algorithm developed by Google (24), which identifies others words used in similar contexts for a given word. Finally, we selected all the tweets that included the words identified by the Word2vec algorithm. Two research assistants then confirmed that only those tweets seeking a suicide pact had been selected before their inclusion in the final data set.

**Figure 1:**
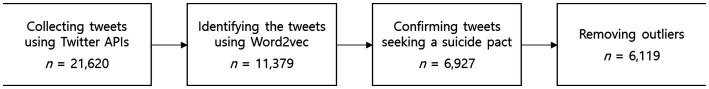
Data preparation flow.

Originally, there were 6,927 tweets seeking others for a suicide pact in the dataset, but tweets by three users were removed as outliers, having posted a large number of tweets (808). Accordingly, the final dataset contained 6,119 tweets. The data preparation flow is illustrated in Figure 1.

#### 2.1.2. Media coverage of celebrity suicides

To retrieve all TV and newspaper reports of celebrity suicides, we used the most popular news aggregator in South Korea, Naver.com. We first searched all news stories containing “suicide” in their titles, from which we then identified stories about the suicide of an entertainment celebrity during the data collection period. We only considered entertainment celebrities, rather than those of other celebrity types, because it has been found that their suicides were more influential on the public (18). There were three entertainment celebrity suicides covered by the media during the data collection period; the second row of Table 1 shows the number of celebrities who died by suicide each month. An entertainment celebrity who committed suicide during the data collection period because he was a suspect of sexual assault was not included in this study, mainly because previous studies suggest that this type of suicide is not regarded as voluntary and leads to few copycat suicides (18).

**Table 1:** Suicide cases by month during the data collection period.

Month	Oct	Nov	Dec	Jan	Feb	Mar	Apr	Total
Celebrity	0	0	1	1	0	1	0	3
Suicide pact	2	3	0	2	5	2	2	16

#### 2.1.3. Media coverage of suicide pact victims

To find reports about suicide pact victims, we first searched all news stories containing “suicide pact” in their titles, from which we identified those stories about the victims. During the data collection period, 16 cases of suicide pacts were covered by the media; the last row of Table 1 shows the number of suicide pacts each month.

#### 2.1.4. The frequency of searching for “suicide pact” or “how to die by suicide”

Data on how frequently people searched for each of two terms, “suicide pact” and “how to die by suicide,” on the most popular Korean search engine, www.naver.com, were collected. The site provides the daily search frequency of a given term per day during the data collection period as a value between 0 and 100 relative to the highest search frequency, given as 100, over the same time.

### 2.2. Regression models

For this study, we constructed three different regression models.

#### 2.2.1. Model 1

The first model aimed to examine how the number of users who posted tweets seeking others to make a suicide pact was associated with the media coverage of celebrity suicides or suicide pacts.


(1) $$\begin{array}{l} \#\text{users\_posting\_suicide\_pac}t_{t}=\\ \beta_{0}+\beta_{1}.\text{whether\_celebrity\_suicide\_last}3\text{day}s_{t}+\\ \beta_{2}.\text{whether\_sui\_pacts\_last}3\text{day}s_{t}+\eta .\text{year\_}\\ \text{effect}s_{t}+\mu .\text{month\_effect}s_{t}+\sigma .\text{day\_effect}s_{t}+\text{erro}r_{t} \end{array}$$

where, *#users_posting_suicide_pact_t_* refers to the number of users who posted tweets seeking others to make a suicide pact on day *t*.

*whether_celebrity_suicide_last3days_t_* refers to whether there was media coverage of celebrity suicides during the past 3 days (As a comparison, we also tested the model using an independent variable reflecting whether there was media coverage of celebrity suicides during the past 7 days. The results indicated that the effect of media coverage declines over time).

*year_effects_t_*, *month_effects_t_*, and *day_effects_t_* are dummy variables reflecting year, month, and weekday of day *t*. These variables were included because it is likely that the number of users posting such tweets varies by year, month, and weekday.

#### 2.2.2. Model 2

The second model aimed to examine how the number of users posting such tweets was associated with the frequency of searching for “how to die by suicide” or “suicide pact.”


(2) $$\begin{array}{l} \#\text{users}:\text{posting}:\text{suicide}:pact_{t}=\beta_{0}+\beta_{1}.\text{search}:fre:\\ \text{how}:{\text{suicide}}_{t}+\beta_{2}.\text{search}:fre:\text{suicide}:{\text{pact}}_{t}+\eta .\text{year}:\\ {\text{effects}}_{t}+\mu .\text{month}:{\text{effects}}_{t}+\sigma .\text{day}:{\text{effects}}_{t}+{\text{error}}_{t} \end{array}$$

where, *search_fre_how_suicide_t_* refers to the frequency of searching for “how to die by suicide” on day *t*.

*search_fre_suicide_pact_t_* reflects the frequency of searching for “suicide pact” on day *t*.

#### 2.2.3. Model 3

The third model aimed to find the relationship between the search frequency for “how to die by suicide” or “suicide pact” and the media coverage of celebrity suicides or suicide pacts.


(3) $$\begin{array}{l} \text{search\_fr}e_{k,t}=\beta_{0}+\beta_{1}.\text{whether\_celebrity\_}\\ \text{suicide\_last}3\text{day}s_{t}+\beta_{2}.\text{whether\_sui\_pacts\_}\\ \text{last}3\text{day}s_{t}+\eta .\text{year\_effect}s_{t}+\mu .\text{month\_}\\ \text{effect}s_{t}+\sigma .\text{day\_effect}s_{t}+\text{erro}r_{t} \end{array}$$

where, *search_fre_k,t_* refers to the frequency of searching for term *k* on day *t*. *k* is either the term “how to die by suicide” or “suicide pact.”

These models were estimated using the OLS estimation method with a robust standard error option.

## 3. Results

### 3.1. Descriptive results

The number of tweets in the final dataset, which was 6,119, indicates that the average number of such tweets per day was 34.0. The number of users, which was based on different screen names on Twitter, in the final data was 2,361. This indicates that on average a user posted 2.59 tweets and 13.1 users posted such tweets per day during the data collection period.

### 3.2. Estimation results of model 1

Table 2 shows the estimation results of Model 1. As shown in Table 2, the number of users posting tweets seeking others to make a suicide pact on a particular day increased when there was media coverage of celebrity suicides during the last 3 days, and the increase was statistically significant. In addition, the number of users posting such tweets was found to be negatively associated with the media coverage of suicide pacts during the last 3 days, albeit statistically insignificant. There tended to be more users per day in 2018 than 2017 and more tweets posted in January than other months.

**Table 2:** Estimation results of Model 1.

Variables	*B*	*SE B*	*p*	*95% CI*
Whether media coverage of celebrity suicide in past 3 days	4.79	2.28	0.037	[0.28, 9.30]
Whether media coverage of suicide pact in past 3 days	−2.27	1.23	0.066	[−4.70, 0.15]
2018	5.61	1.66	0.001	[2.34, 8.87]
February	−1.40	1.73	0.419	[−4.82, 2.02]
March	−8.97	1.69	< 0.001	[−12.30, −5.64]
April	−8.73	1.68	< 0.001	[−12.05, −5.41]
October	0.002	2.08	0.999	[−4.10, 4.10]
November	−2.36	1.74	0.177	[−5.79, 1.07]
December	(Omitted)			
Mon	1.70	1.78	0.341	[−1.81, 5.21]
Tue	0.31	1.75	0.861	[−3.16, 3.77]
Wed	−0.55	1.84	0.764	[−4.18, 3.08]
Thu	0.53	1.81	0.769	[−3.04, 4.10]
Fri	−2.36	1.78	0.186	[−5.87, 1.15]
Sat	−3.06	1.77	0.086	[−6.57, 0.44]
Constant	23.51	1.64	< 0.001	[20.27, 26.75]
*R* ^2^	0.31			
*F(14, 165)*	5.18^**^			

*N* = 180. For the years, months, and the days of week dummy variables, 2017, January, and Sunday were used as the reference, respectively.

### 3.3. Estimation results of model 2

Table 3 shows the estimation results of Model 2. According to Table 3, the search frequency on “how to die by suicide” had a positive and statistically significant association with the number of users posting tweets to seek others for a suicide pact at a 0.05 significance level. On the other hand, although the search frequency for “suicide pact” was positively associated with the dependent variable, it was not statistically significant.

**Table 3:** Estimation results of Model 2.

Variables	*B*	*SE B*	*p*	*95% CI*
Search frequency on “how to die suicide”	0.11	0.06	0.050	[0.00, 0.22]
Search frequency on “suicide pact”	0.04	0.04	0.402	[−0.05, 0.12]
Constant	17.54	3.33	< 0.001	[10.97, 24.11]
*R* ^2^	0.30			
*F(14, 165)*	4.97^**^			

*N* = 180. The results of the dummy variables about year, month, and day effects were omitted mainly due to their similarities to their counterparts in Table 2.

### 3.4. Estimation results of model 3

Table 4 shows the estimation results of Model 3, for which we used two different dependent variables: (1) search frequency on “how to die by suicide,” and (2) search frequency on “suicide pact.” The frequency of searching the term, “how to die by suicide,” was more strongly associated with the media coverage of celebrity suicide than that of suicide pact victims, while the frequency of searching “suicide pact” was more strongly associate with the media coverage of suicide pact victims.

**Table 4:** Estimation results of Model 3.

	*“How to die by suicide”*				*“Suicide pact”*			
Variables	*B*	*SE B*	*p*	*95% CI*	*B*	*SE B*	*p*	*95% CI*
Whether media coverage of celebrity suicide in past 3 days	16.95	2.91	<0.001	[11.20, 22.70]	1.24	3.98	0.756	[−6.61, 9.09]
Whether media coverage of suicide pact in past 3 days	−0.11	1.56	0.945	[−3.20, 2.98]	8.57	2.14	<0.001	[4.35, 12.79]
Constant	43.97	2.09	<0.001	[39.84, 48.09]	38.44	2.85	<0.001	[32.81, 44.08]
*R* ^2^	0.41				0.26			
*F(14, 165)*	8.34^**^				4.25^**^			

*N* = 180. The results of the dummy variables about year, month, and day effects were omitted mainly due to the space constraint.

## 4. Discussion

In this study, we examined whether (or how) media coverage of either celebrity suicides or suicide pacts and the frequency of searching for either “how to die by suicide” or “suicide pact” predicted the number of individuals who posted tweets seeking others to make a suicide pact. We also investigated the association between media coverage of suicides and the search frequency on suicides.

First, we found that the number of users posting tweets seeking others for a suicide pact was positively associated to whether there was media coverage of celebrity suicides at a 0.05 significance level, while its association with media coverage of suicide pacts was statistically insignificant. If the former type of media coverage is assumed to lead individuals to copy the suicidal behavior, the positive association indicates that some of them might want to attempt suicide as part of a suicide pact, and thus post tweets seeking others to make a suicide pact. Prior studies examining the effects of media coverage of a suicide on copycat suicides found positive effects of such media coverage [e.g. (15, 16, 17–19)]. In addition to the findings of these studies, the present study provides new insights regarding the roles of social media between media coverage of a suicide and public copycat suicides. That is, the results of this study suggest that media coverage of a suicide may motivate individuals to seek others for a suicide pact on social media, which is likely to results actual suicides.

On the hand, the insignificant association with the media coverage of suicide pact victims suggests the opposite to the horizontal identification principle, which theorizes that media coverage of suicide pacts is likely to lead more people to copy such behavior. People might want to avoid posting tweets seeking others to make a suicide pact when there is media coverage of suicide pacts, because it is likely that there can be more police investigations into such activity on the Web.

Second, we found that the number of users posting tweets seeking others to make a suicide pact was positively associated with the search frequency on “how to die by suicide” at a 0.05 significance level, while its positive association with the search frequency on “suicide pact” was statistically insignificant. These findings indicate that more of those who searched for the former than the latter were considering committing suicide. Some of those who searched for “how to die by suicide” were likely to find information about suicide pacts and consider ending their lives alongside others, whom they would try to find by posting on Twitter. If the number of individuals posting tweets seeking others for a suicide pact was positively associated with suicide rates in public, this finding also suggests a positive relationship between the frequency of searching suicide methods on the Internet and suicide rates, which was confirmed by numerous prior studies [e.g. (21, 25, 26)]. Meanwhile, such searches tended to increase following media coverage of celebrity suicides, indicating some individuals might consider copying their suicidal behavior.

Further, it is likely that those who searched for “suicide pact” included people who merely wanted to know what a suicide pact was rather than how to seek others to make a suicide pact. For example, when there was media coverage of suicide pacts, then there would be more people who searched the term mainly because to know more about what a suicide pact was. This relationship is supported by the finding of the positive association between the media coverage of suicide pacts and the search frequency on “suicide pact.”

### 4.1. Limitations

This study has some limitations. First, the number of users posting tweets was based on different account information such as screen names and user IDs. However, it is possible that the same person uses different accounts to increase their anonymity, meaning that the number of actual users might be smaller than that used in this study. Second, we used data from South Korea, which might not be generalizable to other countries where people have different attitudes toward celebrities. In South Korea, celebrities are generally idolized and recognized as public figures, meaning it is likely that there are more individuals who identify with celebrities (27). Further, although some control variables were incorporated in the regression models, the results of the regression models do not guarantee a causal relationship between the explanatory and dependent variables. To gain a more accurate understanding of the roles of media coverage and Internet searches in explaining the posting of tweets seeking others for a suicide pact, it is necessary to conduct interviews with those who actually posted such tweets. Additionally, even though we identified tweets seeking others to make a suicide pact, it is not certain that this was the actual intention of all the posts; as noted by some news stories (28), tweets seemingly seeking suicide pacts were actually intended to trap people for criminal purposes. Thus, the actual number of tweets seeking others to make a suicide pact might be smaller than that used in this study. Finally, even though the final dependent variable in this study was the number of tweets soliciting others for a suicide pact, it is unknown whether such tweets led to actual suicides. Thus, it is suggested that future research investigate the relationship between the number of tweets seeking a suicide pact and the variation in suicide rates.

### 4.2. Conclusion

In this study, we found that more people posted tweets seeking others to make a suicide pact following media coverage of celebrity suicides, suggesting that some people attempted to copy such suicidal behavior by means of a suicide pact. To reduce the number of victims of suicide pacts formed through Twitter, therefore, more public effort is needed to monitor such tweets, especially after celebrity suicides are reported. Furthermore, it is important to limit the amount of media coverage and use the media to try and prevent the audience from imitating the suicidal behavior. Even though the focus of this study was on the quantity of media coverage, it is also crucial that media report suicides in an appropriate manner. Media reporting guidelines for a suicide [e.g., (29)] recommend not only limited media coverage of a suicide, but also appropriate reporting of a suicide, including the use of insensible language and omission of method details. As this study also suggests that the search frequency on “how to die by suicide” can predict the number of users seeking others to make a suicide pact, when it increases, the monitoring of tweets for suicide pacts needs to be more thorough. Moreover, the social media company should recognize how its platform can be used to seek others for suicide pacts and try to filter out such tweets. As the results of this study indicate a positive association between media coverage of a suicide and the number of individuals posting tweets seeking a suicide pact, it is recommended that the future study examine whether or how media coverage of a suicide influences an individual’s such posting behavior with other research methods such as interviews. In addition, it is suggested that future research investigate whether tweets seeking a suicide pact lead to actual suicides.

## Data availability statement

The raw data supporting the conclusions of this article will be made available by the authors, without undue reservation.

## Author contributions

SL: Conceptualization, Data curation, Formal analysis, Funding acquisition, Methodology, Project administration, Writing – original draft, Writing – review & editing.
